# Passive Sensing in the Prediction of Suicidal Thoughts and Behaviors: Protocol for a Systematic Review

**DOI:** 10.2196/42146

**Published:** 2022-11-29

**Authors:** Tanita Winkler, Rebekka Büscher, Mark Erik Larsen, Sam Kwon, John Torous, Joseph Firth, Lasse B Sander

**Affiliations:** 1 Institute of Psychology University of Freiburg Freiburg Germany; 2 Medical Psychology and Medical Sociology Faculty of Medicine University of Freiburg Freiburg Germany; 3 Black Dog Institute University of New South Wales Sydney Australia; 4 Beth Israel Deaconess Medical Center Harvard Medical School Boston, MA United States; 5 Division of Psychology and Mental Health Manchester Academic Health Science Centre University of Manchester Manchester United Kingdom

**Keywords:** suicide prediction, passive sensing, review, systematic review, sensors, suicidal thoughts and behaviors, digital markers, behavioral markers

## Abstract

**Background:**

Suicide is a severe public health problem, resulting in a high number of attempts and deaths each year. Early detection of suicidal thoughts and behaviors (STBs) is key to preventing attempts. We discuss passive sensing of digital and behavioral markers to enhance the detection and prediction of STBs.

**Objective:**

The paper presents the protocol for a systematic review that aims to summarize existing research on passive sensing of STBs and evaluate whether the STB prediction can be improved using passive sensing compared to prior prediction models.

**Methods:**

A systematic search will be conducted in the scientific databases MEDLINE, PubMed, Embase, PsycINFO, and Web of Science. Eligible studies need to investigate any passive sensor data from smartphones or wearables to predict STBs. The predictive value of passive sensing will be the primary outcome. The practical implications and feasibility of the studies will be considered as secondary outcomes. Study quality will be assessed using the Prediction Model Risk of Bias Assessment Tool (PROBAST). If studies are sufficiently homogenous, we will conduct a meta-analysis of the predictive value of passive sensing on STBs.

**Results:**

The review process started in July 2022 with data extraction in September 2022. Results are expected in December 2022.

**Conclusions:**

Despite intensive research efforts, the ability to predict STBs is little better than chance. This systematic review will contribute to our understanding of the potential of passive sensing to improve STB prediction. Future research will be stimulated since gaps in the current literature will be identified and promising next steps toward clinical implementation will be outlined.

**Trial Registration:**

OSF Registries osf-registrations-hzxua-v1; https://osf.io/hzxua

**International Registered Report Identifier (IRRID):**

DERR1-10.2196/42146

## Introduction

### Suicide Prevention

Suicide is a common cause of death, resulting in over 700,000 deaths worldwide each year, while the total number of suicide attempts is even higher [1]. Especially among adolescents and young adults, suicide is a leading cause of death [1]. Identification of suicidal thoughts and behaviors (STBs) can support help seeking to prevent suicide attempts [2]. Years of research have been conducted to identify risk factors to meet this demand of health care [3-7]. Yet, a recent systematic review of prediction models for suicide attempts and deaths reported that the predictive validity associated with a positive result for suicide mortality was extremely low (≤0.01 in most models) [8]. Additionally, Franklin and colleagues [4] found that research on suicide risk factors in the past 50 years carries almost no predictive power due to several reasons. First, the prediction of a suicidal event is difficult in general because of the low probability of the occurrence of suicide [2,6]. Second, a major shortcoming of prior investigations was the repeated focus on an identical set of risk factors that have been shown to produce little predictive value for when an attempt might occur. For example, while the first suicidal thoughts or ideas about specific suicide methods often occur years before a suicide attempt, the transfer from ideation to action often occurs within days or hours before the attempt [9]. Third, similar measurement methods were repeatedly used, predominantly questionnaires [4]. However, questionnaires fall short in describing the strong fluctuations in risk factors for suicidal behavior like emotional distress, hopelessness, or suicidal thoughts [10,11], making short-term prediction nearly impossible. In addition, it is difficult to model the complex interactions of different risk factors using questionnaire data [12].

### Passive Sensing

In recent years, promising new ways to identify STBs have emerged. The widespread use of smartphones in everyday life provides a source of data that allows real-time monitoring [13]. Passive sensing is one of many terms that are used to describe the passive collection of behavioral data via smartphones or other wearable devices [14]. Depending on the type of device, different sensors can be used to collect a variety of data points. In general, behavioral (eg, movement via GPS), physiological (eg, heart rate), and social (eg, social media engagement) signals can be measured through the sensors [13]. The objectivity and seamless provision of information over a period of time brings advantages compared to subjective questionnaires at a certain point in time [15,16]. Therefore, research has focused on the potential and feasibility of passive sensing in mental health [16-19]. Machine learning methods can then build predictive models from these huge data sets [20,21].

### Previous Research

Despite a number of unanswered questions and methodological challenges [22], some researchers have explored the potential of passive sensing for mental health. Studies investigating the relationship between passive sensing and psychological symptoms have been conducted [18,23,24]. For example, Zulueta and colleagues [25] found that increased accelerometer activity was correlated with a change in mood disturbances. Furthermore, increased social media use was found to be positively correlated to depressive symptoms [25]. Quantitative variables such as the number of outgoing calls, unique numbers texted, or absolute distance traveled showed a predictive value for depressive symptoms in an investigation by Place and colleagues [26]. Across several studies, there is a promising trend that passive sensing can be reliably used to predict some symptoms or behaviors. However, other studies report less-promising results, which tempers expectations and highlights the need for replication trials to confirm preliminary findings [27,28].

### The Proposed Review

Prior reviews have been conducted to summarize the possibilities and limitations of passive sensing in suicide prediction [15,29,30]. Given the dynamic development of the field, an updated review is needed to observe the latest advances in the field. The proposed systematic review aims to summarize the research on passive sensing in suicide prediction and identify both major advances and obstacles. In particular, we intend to create a better understanding of how current findings may translate into practice. For this purpose, the review will address (1) whether passive sensing data show the ability to improve the prediction of STBs compared to traditional methods of data collection, (2) what the comparative predictive power of different sensor types is, and (3) what analysis methods have been reported in the literature.

## Methods

This protocol is based on the PRISMA-P (Preferred Reporting Items for Systematic Review and Meta-Analysis Protocols) checklist [31].

### Eligibility Criteria

We will include studies that addressed passive data generation via smartphones or wearables in the context of STBs. All individuals regardless of age or gender with any STB (suicidal ideation, suicide attempt, death by suicide) will be included. Studies reporting on nonsuicidal self-injury will be excluded. If studies investigate both STBs and nonsuicidal self-injury, they will be included. Studies will be included regardless of whether or not the participants were receiving treatment. Studies will be eligible if they report results on the association between passive sensing and STBs. In addition, we will include study protocols and conduct a search of international study registries to preview upcoming research. Articles will be translated into English if necessary.

### Search Strategy

A web-based systematic database search will be performed using the following search terms: (mobile sens* OR smart sens* OR smartphone sens* OR passive sens* OR passive monitor* OR sensor OR sensors OR digital phenotyp* OR wearable* OR passive data OR real-time data OR real-world data) AND suicid*. To ensure the sensitivity of the search, the search string was validated by a test set of 7 hand-searched relevant articles (Multimedia Appendix 1 [32-38]). The search string was optimized using an iterative process until a coverage rate of 100% was reached. In July 2022, the search will be conducted via the databases MEDLINE, PubMed, Embase, PsycINFO, and Web of Science (Multimedia Appendix 2). We will perform forward searches via Google Scholar and backward searches in the reference lists. Gray literature will not be searched.

### Selection Process

The selection of relevant articles will be conducted by 2 independent researchers using the online tool Covidence. First, all titles and abstracts resulting from the search will be screened against the eligibility criteria. Second, the full texts of the articles selected in the first step will be obtained and screened in more detail. Disagreements will be resolved in discussion with a third reviewer. Duplicates will be identified and excluded. In the case of multiple reports of the same study, all available data will be reported. All steps of the selection process will be described in detail and will be visualized in a PRISMA (Preferred Reporting Items for Systematic Reviews and Meta-Analyses) flowchart [39].

### Data Extraction

All data will be managed via Covidence, which will be used for the whole selection process. At the end of the process, the 2 independent reviewers will extract information according to a data extraction sheet. The following variables will be extracted from all included articles: authors, publication year, journal, population variables (age, gender, STBs), data collection device (smartphone, smartwatch, smart home, etc), type and frequency of passive data (social media, text messages, screen time, etc), analysis methods (machine learning, predictive models, qualitative techniques, etc), and assessment length. If any important data are missing, the authors will be contacted. Any qualitative or quantitative data describing the predictive ability of passive sensor data will be extracted. Results on the practical relevance and feasibility of passive sensing will be considered as secondary outcomes.

### Risk of Bias Assessment

To assess the risk of bias at the study level, 2 independent reviewers will use the signaling questions of the Prediction Model Risk of Bias Assessment Tool (PROBAST) [40]. According to the tool, 4 domains will be analyzed: participants, predictors, outcome, and analysis. In total, 19 signaling questions will be answered with “yes,” “probably yes,” “no,” “probably no,” or “no information.” Afterward, the level of risk of bias will be estimated. Disagreement between the reviewers will be solved via discussion. If discussion does not lead to agreement, a third reviewer will be consulted.

### Data Analysis

All extracted characteristics of the identified studies will be described narratively. The relevant results will be presented in text form and visualized in tables. If an appropriate number of studies report associations between identical sensor data and a quantitative measure for STBs, we will perform meta-analytic pooling. The meta-analytic pooled correlation will be estimated using a random-effects model with a maximum likelihood estimator. We will treat the heterogeneity (ie, the variability between the studies in terms of methodology and sample characteristics) as random [41]. In this way, we will estimate both the average true effect and the amount of heterogeneity among the true effects [41]. If heterogeneity is zero, the average true effect displays the true effect.

## Results

The selection process started in July 2022. Data extraction started at the beginning of September 2022. Results are expected in December 2022.

## Discussion

The aim of this systematic review is to summarize the potential of passively generated data to predict STBs. Research in this area is new but has developed rapidly in recent years. Consequently, we expect a rather heterogenous set of reports and trial designs. Therefore, one aspect of this review will be to identify key variables that future trials should report in order to increase comparability in future systematic reviews. For example, an extended form of the TRIPOD (Transparent Reporting of a Multivariable Prediction Model for Individual Prognosis or Diagnosis) Checklist [42] for sensing data could be developed. In addition, this review offers a chance to identify valuable predictors to improve the prevention of suicide. It will present an updated summary of existing knowledge in this fast-growing field and exceed prior reviews’ quality through preregistration and by using a systematic approach. Next to predictive values of quantitative sensor data, feasibility aspects of sensing studies will be pointed out as well. Hence, new inspirations for further research regarding methodological possibilities of data collection and study design will be stimulated. At the same time, recent evidence will be critically evaluated in order to create further demands for research that will advance the path to clinical applicability.

## Appendix

Multimedia Appendix 1 The test set of 7 hand-searched relevant articles.

Multimedia Appendix 2 Database search strings.

## Abbreviations

PRISMA: Preferred Reporting Items for Systematic Reviews and Meta-Analyses
PRISMA-P: Preferred Reporting Items for Systematic Review and Meta-Analysis Protocols
PROBAST: Prediction Model Risk of Bias Assessment Tool
STB: suicidal thoughts and behavior
TRIPOD: Transparent Reporting of a Multivariable Prediction Model for Individual Prognosis or Diagnosis

## Data Availability

All study data will be made publicly available on the Open Science Framework website.
