# Supplementary material for: Passive Sensing in the Prediction of Suicidal Thoughts and Behaviors: Protocol for a Systematic Review
Source: JMIR Res Protoc. 2022 Nov 29;11(11):e42146. doi: 10.2196/42146 (PMC9748797; doi:10.2196/42146)
Supplement: Multimedia Appendix 1 [file resprot_v11i11e42146_app1.docx]

Multimedia Appendix 1. Test set of the 7 hand-searched relevant articles

| **Authors** | **Year** | **Journal** | **Title + doi** |
| --- | --- | --- | --- |
| Sheridan et al. | 2021 | Psychiatry Investigation | Heart Rate Variability and Its Ability to Detect Worsening Suicidality in Adolescents: A Pilot Trial of Wearable Technology  <https://doi.org/10.30773/pi.2021.0057Print> |
| Kleiman et al. | 2021 | Translational Psychiatry | Can passive measurement of physiological distress help better predict suicidal thinking?  <https://doi.org/10.1038/s41398-021-01730-y> |
| Haines-Delmont et al. | 2020 | JMIR MHEALTH AND UHEALTH | Testing Suicide Risk Prediction Algorithms Using Phone Measurements With Patients in Acute Mental Health Settings: Feasibility Study  <https://doi.org/10.2196/15901> |
| Coyne et al. | 2021 | Journal of Youth and Adolescence | Suicide Risk in Emerging Adulthood: Associations with Screen Time over 10 years  <https://doi.org/10.1007/s10964-020-01389-6> |
| Sels et al. | 2021 | Frontiers in Psychiatry \| | SIMON: A Digital Protocol to Monitor and Predict Suicidal Ideation  <https://doi.org/10.3389/fpsyt.2021.554811> |
| Berrouiguet et al. | 2019 | BMC Psychiatry | Combining mobile-health (mHealth) and artificial intelligence (AI) methods to avoid suicide attempts: the Smartcrises study protocol  <https://doi.org/10.1186/s12888-019-2260-y> |
| Bruen et al. | 2020 | JMIR MENTAL HEALTH | Exploring Suicidal Ideation Using an Innovative Mobile App-Strength Within Me: The Usability and Acceptability of Setting up a Trial Involving Mobile Technology and Mental Health Service Users <https://doi.org/10.2196/18407> |
