# Supplementary material for: Passive Sensing in the Prediction of Suicidal Thoughts and Behaviors: Protocol for a Systematic Review
Source: JMIR Res Protoc. 2022 Nov 29;11(11):e42146. doi: 10.2196/42146 (PMC9748797; doi:10.2196/42146)
Supplement: Multimedia Appendix 2 [file resprot_v11i11e42146_app2.docx]

| Multimedia Appendix 2. Table showing the search string for each database. | | | | | |
| --- | --- | --- | --- | --- | --- |
|  | **Web of Science** | **PubMed** | **MEDLINE** | **PsycINFO** | **Embase** |
| S1 | mobile sens* | mobile sens* | mobile sens* | mobile sens* | mobile sens* |
| S2 | smart sens* | smart sens* | smart sens* | smart sens* | smart sens* |
| S3 | smartphone sens* | smartphone sens* | smartphone sens* | smartphone sens* | smartphone sens* |
| S4 | passive sens* | passive sens* | passive sens* | passive sens* | passive sens* |
| S5 | passive monitor* | passive monitor* | passive monitor* | passive monitor* | passive monitor* |
| S6 | TI sensor OR AB sensor | TI sensor OR AB sensor | TI sensor OR AB sensor | TI sensor OR AB sensor | TI sensor OR AB sensor |
| S7 | TI sensors OR AB sensors | TI sensors OR AB sensors | TI sensors OR AB sensors | TI sensors OR AB sensors | TI sensors OR AB sensors |
| S8 | digital phenotyp* | digital phenotyp* | digital phenotyp* | digital phenotyp* | digital phenotyp* |
| S9 | wearable* | wearable* | wearable* | wearable* | wearable* |
| S10 | passive data | passive data | passive data | passive data | passive data |
| S11 | real-time data | real-time data | real-time data | real-time data | real-time data |
| S12 | real-world data | real-world data | real-world data | real-world data | real-world data |
| S13 | S1 OR S2 OR S3 OR S4 OR S5 OR S6 OR S7 OR S8 OR S9 OR S10 OR S11 OR S12 | S1 OR S2 OR S3 OR S4 OR S5 OR S6 OR S7 OR S8 OR S9 OR S10 OR S11 OR S12 | S1 OR S2 OR S3 OR S4 OR S5 OR S6 OR S7 OR S8 OR S9 OR S10 OR S11 OR S12 | S1 OR S2 OR S3 OR S4 OR S5 OR S6 OR S7 OR S8 OR S9 OR S10 OR S11 OR S12 | S1 OR S2 OR S3 OR S4 OR S5 OR S6 OR S7 OR S8 OR S9 OR S10 OR S11 OR S12 |
| S14 | suicid* | suicid* | suicid* | suicid* | suicid* |
| S15 | S13 AND S14 | S13 AND S14 | S13 AND S14 | S13 AND S14 | S13 AND S14 |
